# Relationship Between Old-Aged Preferences Regarding Various Types of Physical Activity and Chronic Disease Status: A Cross-Sectional Study in Shanghai, China

**DOI:** 10.3389/fpubh.2022.865328

**Published:** 2022-03-24

**Authors:** Xiaojing Huang, Wenqing Zhu, Xiang Gao, Dehua Yu, Hua Jin, Jiaoling Huang, Wenya Yu, Yipeng Lv, Liang Zhou, Ning Chen, Yan Yang, Zhaoxin Wang, Jianwei Shi

**Affiliations:** ^1^School of Management, Xuzhou Medical University, Xuzhou, China; ^2^Shanghai Municipal Center for Disease Control and Prevention, Shanghai, China; ^3^School of Public Health, Shanghai Jiao Tong University School of Medicine, Shanghai, China; ^4^Department of General Practice, Yangpu Hospital, Tongji University School of Medicine, Shanghai, China; ^5^Academic Department of General Practice, Tongji University School of Medicine, Shanghai, China; ^6^Shanghai General Practice and Community Health Development Research Center, Shanghai, China; ^7^School of Economics and Management, Tongji University, Shanghai, China; ^8^Department of Social Medicine and Health Management of School of Public Health, Shanghai Jiao Tong University School of Medicine, Shanghai, China

**Keywords:** physical activity, older adults, chronic disease, moderate-intensity physical activity, housework-related physical activity, transport-related physical activity

## Abstract

**Background:**

A lack of physical activity (PA) is a threat to public health. However, for the elderly, most PA abilities are limited. By focusing on the types and intensity of PA that the elderly can bear, this study aimed to reveal whether preferences regarding types of PA (including housework, transportation, and recreational activities) and their intensity were associated with health status. The main forms of PA include shopping, cooking, cleaning, walking, cycling, various fitness activities and other activities with a certain intensity.

**Methods:**

Surveillance data on chronic diseases and their risk factors were collected from one district of Shanghai in 2017-2018. A Kish table was used for sampling 500 older adults, including the diagnosed group (chronic diseases diagnosed by physicians, *n* = 119), the abnormal group (not diagnosed but abnormal indicators detected in this investigation, *n* = 287) and the healthy group (*n* = 94). Multiple regressions were used to test the relationship between the various types, durations and intensities of PA the elderly individuals participated in and their health status.

**Results:**

All three groups included a large proportion of older adults who participated in housework- and transport-related PA. The diagnosed group had the largest proportion (63.06% for housework-related PA; 87.39% for transport-related PA) and median minutes (17.14 min of housework-related PA per day; 30.00 min of transport-related PA per day). The diagnosed group had more metabolic equivalents (METs) of moderate-intensity PA than the two other groups (H = 33.01, *P* < 0.01), and more people met the WHO recommendation (χ^2^ = 34.71, *P* < 0.01). Diagnosis was associated with performing housework- and transport-related PA and moderate-intensity PA and with meeting the WHO's recommendation. Higher education levels were a positive factor for elderly individuals to participate in PA.

**Conclusions:**

Transportation and housework activities are good targets for increasing PA in older adults. Diagnosis is associated with older adults' more PA.

## Introduction

Physical activity (PA) has significant health benefits and contributes to the prevention and treatment of non-communicable diseases (NCDs), such as diabetes, cardiovascular disease, hypertension, and some cancers (1–4). Patients with chronic diseases need more regular PA than healthy residents to increase the effect of treatment and avoid adverse disease consequences (5, 6). According to the World Health Organization (WHO), PA is regarded as an important strategy for preventing or treating some chronic diseases and refers to any part of work, transport (walking, wheeling and cycling), sport and leisure, as well as every day and household tasks (7). Elderly individuals were less likely to conduct the exercises but more likely to perform moderate-intensity PA (8).

This is because increased age involves declining PA and increased chronic disease risk (9). A study of older European people's PA showed that nearly half of the elderly population was not adequately active (10). Another study that examined the PA levels of adults over 50 in 6 low- and middle-income countries reported that of the 34,129 respondents, only 23.5% met the recommended PA level (11). With a further increase in age, the amount of PA will gradually decrease. The recent update to the US National Health Interview Survey provides sobering evidence indicating that age continues to be a prominent factor in engaging in recommended aerobic PA levels. Younger adults (18–24 years) report the highest compliance rate (62.2%, CI 59.33–65.05%), while older adults (≥75 years) report the lowest compliance rate (30.2%, CI 27.9–32.61%) (12).

The worldwide epidemic of chronic diseases is strongly linked to population aging (13). A cohort study from China showed that one-third of Chinese adults had hypertension, and the prevalence increased with age (from 12.6% at 35–39 years of age to 58.4% at 70–74 years of age) (14). Elderly individuals may suffer from chronic diseases accompanied by physical inactivity. Not meeting the WHO recommendations of PA (at least 600 MET-minutes per week) is associated with obesity, diabetes, hypertension and metabolic syndrome (15). Therefore, the health status of elderly people is also an important factor in their participation in PA.

Obviously, not every type of PA is suitable for elderly individuals or elderly individuals with chronic diseases. This raises an interesting question that considers elderly people's PA ability: what PAs are preferred by elderly individuals? Currently, guidelines predominantly focus on healthy age-stratified target groups of children and adolescents, adults, and older adults, but the target group of individuals with NCDs has received scant consideration (16). Studies have shown that the types of PA most popular among older adults were consistently of moderate intensity (walking, gardening, golf, low-impact aerobic activities) compared with those of younger adults (17, 18).

Shanghai, a metropolitan city, was the first city in China to enter the aging society and is a large city with the highest degree of aging in China (19). In 2018, it had 14.6 million registered residents, including an estimated 5.02 million elderly people who were over 60 years old (20). Because of the rapidly aging population, Shanghai began to explore community elderly care services in 2000. The development of community services includes improving the living environment of elderly people, facilitating their daily travel, enriching their cultural life, and expanding their social participation. The construction of a supportive environment in Shanghai is also developing. By the end of 2018, the total length of fitness trails and cycling trails for citizens in Shanghai was 1,415 km, including 240 km of cycling trails and 671 km of greenways. The city has built 16,307 community fitness points, 2,208 citizen courts, 84 community citizen fitness centers, 35 citizen swimming pools, 181 citizen gyms, and many sports fitness facilities to achieve full coverage of the community (21). Compared with 2017, the proportion of people aged 60 and over who often take part in PA has increased (21). However, the statistics do not show the health status of older people who increase their PA. In Shanghai, where the proportion of elderly people who suffer from chronic diseases is 22.52~62.58%, it is particularly important to analyse the PA preferences of elderly individuals with chronic diseases. Identifying the preferences of elderly individuals for PA may help in designing community guidance for PA for elderly residents.

This study aimed to reveal whether resident's preferences regarding types of PA (including activities at work, transportation, recreational activities) and their duration and intensity were associated with resident's health status. It is believed that this study will provide information to improve the guidelines for elderly people.

## Methods

### Data Source and Data Screening

The data came from the Shanghai Centres for Disease Control and Prevention's Regular Monitoring of Chronic Diseases in Yangpu District from October 2017 to March 2018. This survey included 1,004 residents from 240 households in all 12 community streets in Yangpu District, Shanghai. Twenty households were randomly selected from each street. A Kish table was used for sampling, and a household survey was conducted. The questionnaire was administered by professional investigators, who input the investigation results into the information collection system at the same time as inquiry. In addition to answering the questionnaire, the selected people received a unique bar code and went to a designated medical institution to participate in the physical examination, which included height, weight, blood pressure, and blood and urine tests. The examination results were recorded in the body measurement table and entered into the database by the investigators. This analysis was approved by the research ethics committee of Tongji University (ref: LL-2016-ZRKX-017).

The survey was divided into three sections: i) basic personal information, including age, sex, education level, marital status, and occupation; ii) personal PA habits, including the self-reported frequency of PA in a typical week and the self-reported cumulative minutes of PA per day; and iii) personal health status: the body mass index (BMI), fasting glucose, glycosylated hemoglobin, total cholesterol (TC), high-density lipoprotein (HDL), low-density lipoprotein (LDL), triglycerides (TG), urinary protein-to-creatinine ratio, aSBP and aDBP were tested in a health institution; the self-reported history of chronic diseases diagnosed by doctors. In part ii, the data were collected using the Global Physical Activity Questionnaire (GPAQ) (22). In the GPAQ, PA was divided into work (moderate- and vigorous-intensity), transportation and recreational activities (moderate- and vigorous-intensity).

In total, 1,004 people were invited to participate, and 28 people whose physiological indicators were missing were eliminated (the effective response rate was 97.21%). If an individual had a disease that may cause movement restriction, such as heart disease, lung disease, or cerebral infarction, their records were eliminated. Those who were under 60 years old and those over 60 who were still working were also eliminated. Finally, a total of 500 older adults were included, of whom 119 had self-reported chronic diseases diagnosed by doctors (diagnosed group), 287 had some abnormal indicators on our survey but did not have a doctor's diagnosis since the indicators did not support disease status (abnormal group), and 94 were healthy (healthy group).

### Measurements

According to the *GPAQ Analysis Guide* (22), we calculated the total minutes spent on all types of PA per day and the minutes spent on 3 types of activities (housework-, transport-, and recreation-related) per day. Transport-related activity included transportation necessary for commuting, shopping and field work but did not include necessary transportation at work. For example, a courier's riding during delivery is not a transport-related activity, although his or her commute is a transport-related activity. Recreation-related activities included sports and exercises not for the purpose of competition, which refers specifically to activities that occur frequently, not occasionally.

According the guideline, metabolic equivalents (METs) are commonly used to express the intensity of PA. One MET is defined as the energy cost of sitting quietly and is equivalent to a caloric consumption of 1 kcal/kg/h. When calculating a person's overall energy expenditure using GPAQ data, 4 METs are assigned to time spent in moderate activities and 8 METs are assigned to time spent in vigorous activities. The minimum time that adults need in order to benefit from PA is at least 150–300 min of moderate-intensity PA, at least 75–150 min of vigorous-intensity PA, or an equivalent combination of moderate- and vigorous-intensity activity throughout the week (7, 23). We select the lowest value and calculated the cut-off point of METs as 600.

### Outcome Variables

We examined the binary outcome variables of i) performing various types of PA or not (did PA, 1; did no PA, 0) and ii) meeting WHO recommendations on PA for health or not (MET- minutes/week ≥ 600,1; MET- minutes/week <600,0) and examined the continuous outcome variable of total minutes spent on various types of PA per day.

### Independent Variables

The demographic variables in the data were age, sex, education, marital status, and occupation. According to the WHO statistics (24), age was divided into four categories: 60–64, 65–69, 70–74 and ≥75 years old. Education level was divided into primary school or below, junior high school, senior high school, and bachelor's degree or above. BMI was grouped as <18.5, 18.5–23.9, and >23.9.

### Statistical Analysis

Basic demographic characteristics and binary outcome variables are presented as percentages for each group. Continuous outcome variables of the duration of various PAs are presented as medians. Statistical significance was determined by Pearson's chi-square test or Kruskal-Wallis H test. GraphPad Prism 8 (GraphPad Software, San Diego, CA, USA) was used to draw a scatter bar chart of minutes spent on PA per day. Logistic regression models were constructed to analyse the relationship between the types of various PAs and independent variables (models 1 to 3 and models 7 to 9). Linear regression models were used to analyse the relationship between the continuous outcome variables and independent variables (models 4 to 6 and models 10 to 12). All statistical tests were conducted using Stata 14 (StataCorp LLC, College Station, TX, USA). A *p-value* below 0.05 was considered statistically significant.

## Results

### Descriptive and Somatotype Statistics

As shown in Table 1, the proportion of each group aged 60–69 was 59.70% and above. There were more women (55.50–61.70%) than men (38.30–44.50%). The diagnosis group had a higher proportion of bachelor's degrees or above (23.50%), and the difference in educational background distribution was statistically significant (*P* < 0.01). The proportion of married older adults was over 90.00%. The three groups had a higher proportion of normal BMI and overweight, and the two proportions were close in the diagnosed and healthy groups. The differences in the proportions of age group, sex, marital status and BMI level in the three groups were not statistically significant.

**Table 1:** Descriptive statistics of all included participants (*N* = 500, %).

	**Diagnosed group** **(*N* = 119)**	**Abnormal group** **(*N* = 287)**	**Healthy group** **(*N* = 94)**	**χ^2^**	***P-*value**
**Age (years)**
60–64	35.30	42.50	38.30	10.81	>0.05
65–69	24.40	29.30	27.70		
70–74	16.80	11.50	21.30		
≥75	23.50	16.70	12.80		
**Sex**
Male	44.50	40.40	38.30	0.94	>0.05
Female	55.50	59.60	61.70		
**Education**
Primary school or below	5.90	12.50	10.60	40.28	**<0.01**
Junior high school	37.80	42.50	31.90		
Senior high school	32.80	41.10	43.60		
Bachelor's degree or above	23.50	3.80	13.80		
**Marital status**
Unmarried	5.90	5.20	8.50	1.36	>0.05
Married	94.10	94.80	91.50		
BMI*
<18.5	1.70	1.00	3.30	6.76	>0.05
18.5–23.9	49.60	41.10	50.50		
>23.9	48.70	57.80	46.20		

*The BMI index of three people in the healthy group was missing. Bold numbers indicate that the differences were statistically significant*.

### Types of PA

**Figure 1:**
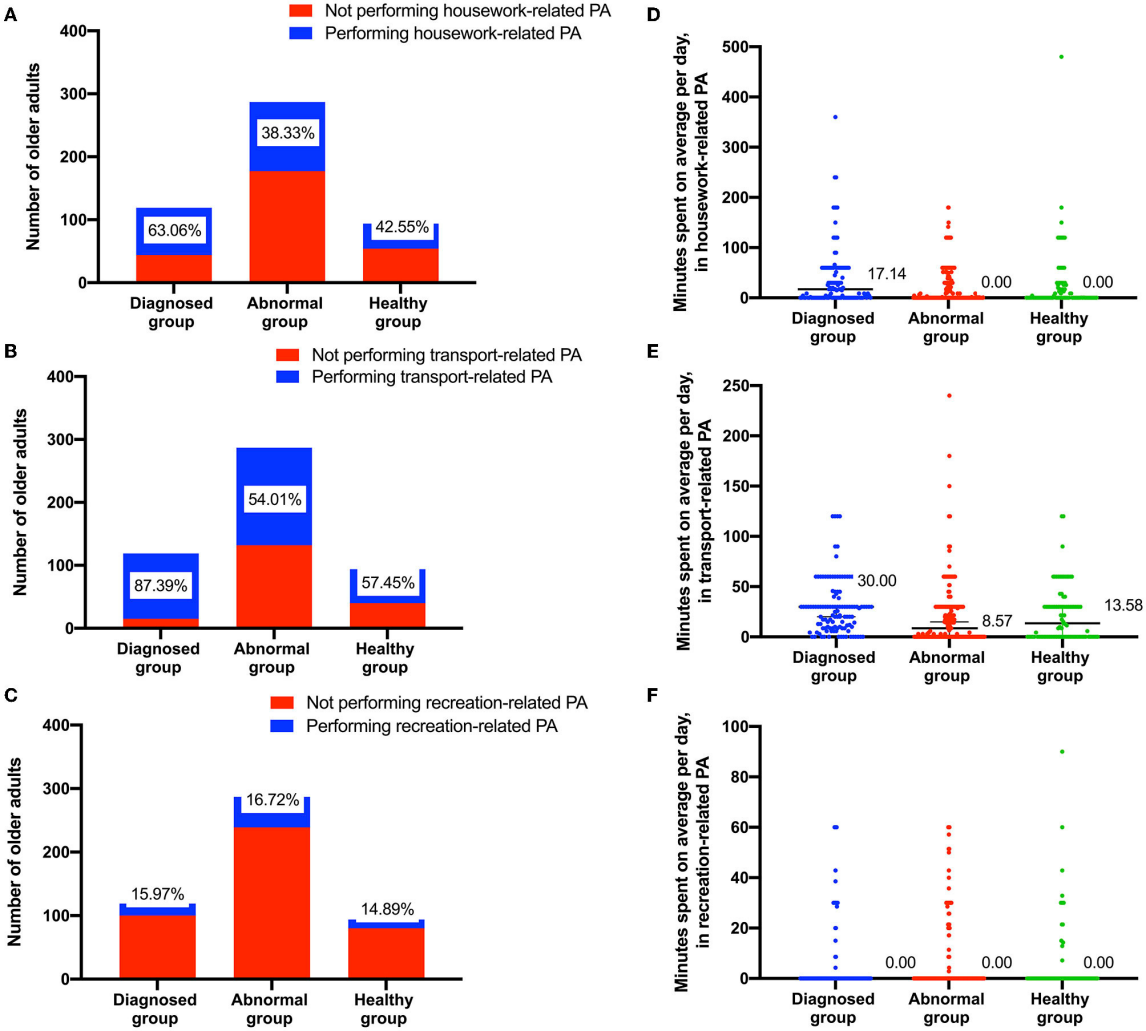
Elderly individuals engaged in three types of PA in three health status groups. **(A–C)** count the number and proportion of older adults participating in the specific type of PA. **(D–F)** show the minutes each older adults spent on the specific type of PA.

Figures 1A–C shows that the proportion of older adults in the diagnosed, abnormal and healthy groups who performed housework-related PA was 63.06, 38.33, and 42.55%, respectively (χ^2^ = 21.01, *P* < 0.01), while the proportion of older adults who performed transport-related PA was 87.39, 54.01, and 57.45%, respectively, in the three groups (χ^2^ = 41.37, *P* < 0.01). The proportion of recreation-related PA was lower than 17%, and the difference among the three groups was not statistically significant (χ^2^ = 0.18, *P* > 0.05). Figures 1D–F shows the minutes older adults spent on housework-, transport- and recreation-related PA. The median time for housework-related PA was 17.14 min per day in the diagnosed group, which was more than the other groups (median = 0.00 min for abnormal group; median = 0.00 min for healthy group) (H = 19.36, *P* < 0.01). A median of 0 does not mean that everyone did not participate in housework-related PA but that more than half of people spent 0 min on housework-related PA. The median times of transport-related PA were 30.00, 8.57, and 13.58 min, respectively, in the three groups (H = 28.00, *P* < 0.01). The older adults in the three groups spent little time on recreational PA, and the difference among the three groups was not statistically significant (H = 0.18, *P* > 0.05).

### Intensity of PA

**Figure 2:**
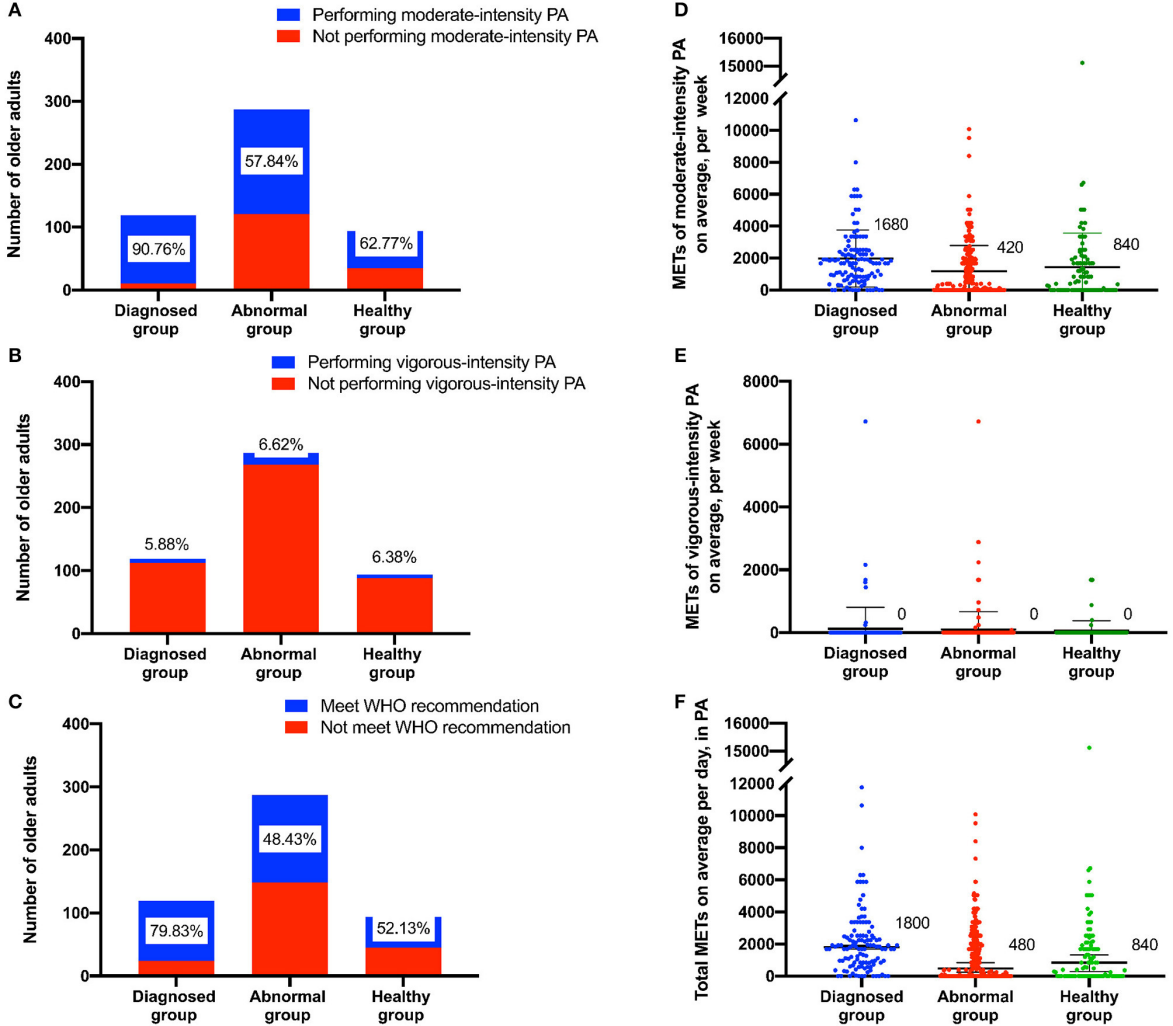
Elderly individuals performing moderate- or vigorous-intensity PA in the three health status groups. **(A,B)** count the number and proportion of older adults participating in the different intensity of PA. **(C)** counts the number and proportion of older adults who met the WHO's recommended level. **(D)** shows the METs of moderate-intensity PA. **(E)** shows the METs of vigorous-intensity PA. **(F)** shows the total METs.

Figures 2A–C shows the proportion of participation in moderate- and vigorous-intensity PA and the proportion of meeting WHO recommendations. A total of 90.76% of older adults in the diagnosed group performed moderate-intensity PA, which was larger than the other two groups (χ^2^ = 41.74, *P* < 0.01). The proportion of vigorous-intensity PA was lower than 7%, and the difference among the three groups was not statistically significant (χ^2^ = 0.07, *P* > 0.05). The diagnosed group also had the largest proportion (79.83%) of older adults who met the WHO's recommendation for PA per week (χ^2^ = 34.71, *P* < 0.01). Figures 2D–F shows the METs of participation in moderate-, vigorous-intensity and total PA. The METs median of moderate-intensity PA per week was 1,680 in the diagnosed group, which was higher than the other two groups (H = 33.01, *P* < 0.01). The median MET of vigorous-intensity PA in the three groups was 0.00, and the difference among the three groups was not statistically significant (H = 0.08, *P* > 0.05). The total METs of PA in the diagnosed group was 1,800, higher than the other two groups (H = 28.85, *P* < 0.01).

### Multiple Regression Analysis of the Types of PA

Table 2 shows that older adults in the abnormal and healthy groups were associated with housework-related PA (OR = 0.39, *P* < 0.01 for the abnormal group; OR = 0.47, *P* < 0.05 for the healthy group in model 1) and transport-related PA (OR = 0.20, *P* < 0.01 for the abnormal group; OR = 0.21, *P* < 0.01 for the healthy group in model 2) more than those in the diagnosed group. Performing PA was associated with higher education levels in models 1 to 3. However, the abnormal and healthy groups were not associated with recreation-related PA (OR = 1.34, *P* > 0.05 for the abnormal group; OR = 0.96, *P* > 0.05 for the healthy group in model 3). The older adults in the diagnosed group spent more minutes on housework-related PA (beta = −0.19, *P* < 0.01) and transport-related PA (beta = −0.16, *P* < 0.05) than the older adults in the abnormal group but not more than the older adults in the healthy group. The association was not statistically significant in model 6. The minutes older adults spent on the three types of PA were associated with higher education levels in models 4 to 6.

**Table 2:** Multiple regression of three types of PA.

	**Model 1**		**Model 2**		**Model 3**		**Model 4**		**Model 5**		**Model 6**	
	**Performing housework-related PA or not**		**Performing transport-related PA or not**		**Performing recreation-related PA or not**		**Minutes of housework-related PA per day**		**Minutes of transport-related PA per day**		**Minutes of recreation-related PA per day**	
	**OR**	***P-*value**	**OR**	***P-*value**	**OR**	***P-*value**	**Beta**	***P-*value**	**Beta**	***P-*value**	**Beta**	***P-*value**
**Group**												
Diagnosed group	Reference						Reference					
Abnormal group	0.39	**<0.01**	0.20	**<0.01**	1.34	0.36	−0.19	**<0.01**	−0.16	**<0.05**	0.04	0.47
Healthy group	0.47	**<0.05**	0.21	**<0.01**	0.96	0.91	−0.10	0.06	−0.08	0.14	−0.01	0.93
**Age (years)**												
60–64	Reference						Reference					
65–69	1.38	0.18	1.25	0.37	1.22	0.52	0.02	0.73	0.03	0.56	0.02	0.65
70–74	1.26	0.43	0.89	0.70	1.80	0.10	0.01	0.90	−0.05	0.27	0.05	0.28
≥75	1.70	0.06	1.34	0.35	0.60	0.22	0.03	0.51	0.13	**<0.05**	−0.07	0.17
**Sex**												
Male	Reference						Reference					
Female	1.43	0.07	1.16	0.48	0.95	0.84	0.14	**<0.01**	0.05	0.24	0.00	0.97
**Education**												
Primary school or below	Reference						Reference					
Junior high school	5.72	**<0.01**	5.18	**<0.01**	10.57	**<0.05**	0.22	**<0.01**	0.32	**<0.01**	0.16	**<0.05**
Senior high school	5.00	**<0.01**	5.78	**<0.01**	13.05	**<0.05**	0.20	**<0.05**	0.21	**<0.01**	0.22	**<0.01**
Bachelor's degree or above	5.16	**<0.01**	12.12	**<0.01**	22.40	**<0.01**	0.07	0.26	0.17	**<0.01**	0.22	**<0.01**
**Marital status**												
Unmarried	Reference						Reference					
Married	0.88	0.76	0.47	0.11	0.48	0.16	−0.06	0.16	−0.08	0.08	−0.04	0.40
**BMI**												
<18.5	Reference						Reference					
18.5~23.9	1.34	0.70	0.73	0.72	0.36	0.25	−0.03	0.85	−0.04	0.83	0.01	0.97
>23.9	1.08	0.92	0.67	0.65	0.44	0.34	−0.08	0.67	−0.08	0.63	0.01	0.94

*Bold numbers indicate that the differences were statistically significant*.

### Multiple Regression Analysis of the Intensity of PA

The results of the multiple regression (Table 3) showed that older adults in the diagnosed group were more likely to participate in moderate-intensity PA (OR = 0.16, *P* < 0.01 for the abnormal group; OR = 0.19, *P* < 0.05 for the healthy group in model 1) and meet WHO recommendations (OR = 0.27, *P* < 0.01 for the abnormal group; OR = 0.30, *P* < 0.01 for the healthy group in model 3). Moderate-intensity PA and meeting WHO recommendations were associated with higher education levels in models 7 and 9. More METs of moderate-intensity PA were associated with healthy status (beta = −0.20, *P* < 0.01 for the abnormal group; beta=-0.11, *P* < 0.05 for the healthy group) in model 10, as in model 12. Females in the diagnosed group (beta = 0.13, *P* < 0.05) with higher education levels (beta = 0.32, *P* < 0.01 for junior high school; beta = 0.21, *P* < 0.01 for senior high school; beta = 0.21, *P* < 0.01 for bachelor's degree or above) reported more METs of moderate-intensity PA, as did model 12.

**Table 3:** Multiple regression of the intensity of PA and meeting WHO recommendations.

	**Model 7**		**Model 8**		**Model 9**		**Model 10**		**Model 11**		**Model 12**	
	**Performing moderate-intensity PA or not**		**Performing vigorous-intensity PA or not**		**Meeting WHO recommendation or not**		**Mets of moderate-intensity PA per week**		**Mets of vigorous-intensity PA per week**		**Mets of PA per week**	
	**OR**	***P-*value**	**OR**	***P-*value**	**OR**	***P-*value**	**Beta**	***P-*value**	**Beta**	***P-*value**	**Beta**	***P-*value**
**Group**												
Diagnosed group	Reference						Reference					
Abnormal group	0.16	**<0.01**	1.31	0.58	0.27	**<0.01**	−0.20	**<0.01**	−0.01	0.93	−0.19	**<0.01**
Healthy group	0.19	**<0.01**	1.38	0.59	0.30	**<0.01**	−0.11	**<0.05**	−0.03	0.60	−0.11	**<0.05**
**Age (years)**												
60–64	Reference						Reference					
65–69	1.13	0.64	1.59	0.31	1.34	0.22	0.02	0.65	0.05	0.29	0.04	0.45
70–74	1.00	1.00	1.64	0.37	0.96	0.90	−0.01	0.85	0.01	0.92	−0.01	0.89
≥75	1.43	0.26	0.72	0.60	2.10	**<0.05**	0.07	0.14	−0.04	0.47	0.06	0.24
**Sex**												
Male	Reference						Reference					
Female	1.08	0.71	0.50	0.07	1.22	0.34	0.13	**<0.05**	0.00	1.00	0.12	**<0.05**
**Education**												
Primary school or below	Reference						Reference					
Junior high school	6.20	**<0.01**	1.00	0.99	7.69	**<0.01**	0.32	**<0.01**	0.10	0.20	0.34	**<0.01**
Senior high school	5.74	**<0.01**	0.51	0.26	7.02	**<0.01**	0.27	**<0.01**	0.07	0.36	0.28	**<0.01**
Bachelor's degree or above	13.93	**<0.01**	1.00	-	9.67	**<0.01**	0.16	**<0.05**	0.07	0.26	0.18	**<0.01**
**Marital status**												
Unmarried	Reference						Reference					
Married	0.59	0.27	1.00	-	0.56	0.20	−0.10	**<0.05**	0.03	0.49	−0.08	0.07
**BMI**												
<18.5	Reference						Reference					
18.5~23.9	0.91	0.92	0.18	0.15	1.44	0.65	−0.05	0.79	0.03	0.86	−0.03	0.85
>23.9	0.86	0.86	0.35	0.37	1.08	0.93	−0.10	0.59	0.03	0.89	−0.08	0.64

*Bold numbers indicate that the differences were statistically significant*.

## Discussion

In this study, we were concerned about the preference of elderly patients with chronic diseases for three types of PA. We found that elderly individuals, regardless of whether they suffered from chronic diseases, spent the longest average time on transport-related PA per day, which is a type of PA they enjoy. Elderly people were likely to prefer slower-paced PA compared to younger adults, who preferred fast-paced PA (25). Transportation can address this preference. Shanghai has built sufficient pedestrian, cycling and greenway areas for elderly people, and retired elderly individuals have sufficient space for transport-related PA. According to the *Shanghai Resident Fitness Development Report 2018*, Shanghai residents enjoy walking and cycling. Aging is associated with a progressive loss of bone-muscle mass and strength (26, 27). Walking is an important way to maintain muscle function (28, 29) and reduce the risk of related diseases. Another PA that occupies a large amount of time is housework activities, which are unavoidable. One of the characteristics of aging in Shanghai is that there are many families in which all members are over 60 years old. By the end of 2018, the number of elderly people in “families with only elderly” in Shanghai reached 1.33 million (30). In addition to the need for care, housework is done by elderly people themselves. In addition, under the influence of fast-paced urban life, even in families with young people, housework is performed by elderly family members as long as their health allows. The preference of the elderly for PA may thus be a passive choice.

Studies have confirmed a positive association between time devoted to housework activities, total housework and health status among elderly men and women (31) that can help prevent the deterioration of disability (32). This result suggests that health guidance can indicate the preferred items of elderly individuals who are physically inactive and diagnosed with chronic diseases.

We were also concerned about whether older adults with a diagnosis of chronic diseases are active in PA. This study found that the proportion of older adults with chronic diseases who participated in both housework- and transport-related PA was higher than the other two groups; they also spent more time on the two types of PA than other groups. The preference of older adults in the abnormal group was similar to those in the healthy group and lower than the diagnosed group. Unlike the abnormal group, patients with chronic diseases were usually instructed by doctors to increase their activity. Many studies have confirmed the effectiveness of regular physical activity in the primary and secondary prevention of several chronic diseases (e.g., cardiovascular disease, diabetes, cancer, hypertension, obesity, depression and osteoporosis) and premature death (33–37). Publicity, notification and health education for patients with chronic diseases are listed in the catalog of community health service projects in Shanghai. However, elderly individuals in the abnormal group may ignore their own health, and they are unable to obtain more information and support from the outside world. Therefore, identifying older adults in the early stage of chronic diseases may be a first step in implementing PA strategies in the community. These differences in preference among different groups may be related to diagnosis information. Studies have shown that a diagnosis can act as an important catalyst to prompt health behavior changes (38, 39). Because this paper is a cross-sectional study, it is impossible to determine whether the diagnostic information is related to the increase of PA.

This study shows that nearly 80% of the older adults in the diagnosed group meet the WHO's recommendation (7, 23), and only approximately 50% of the other two groups meet this standard. The WHO's recommendation is the minimum PA level needed to maintain health. In 2018, the per capita consumption expenditure in Shanghai was 43,351 yuan (20), while the per capita monthly pension for enterprise retirees was 3,851 yuan (46,212 yuan per year), which was received by 4.49 million retirees (89.33% of the elderly population) (40). Therefore, under normal circumstances, elderly people do not need to continue to engage in livelihood activities after retirement. The lack of PA may be caused by the personal fitness literacy of elderly people. In 2018, the fitness literacy scores of citizens aged 50 and above were significantly lower than the scores of those under 50 (21). However, this phenomenon is not unique to China. In the UK, despite much publicity, the overwhelming majority of elderly people still do not meet the minimum levels (41). Although PA is an intervention strategy to improve quality of life, manage symptoms and extend survival, its popularization has great resistance in practice (42). Some studies suggest that necessary interventions may change the activities of the elderly, such as health apps (43) and social support (44).

We also observed that education has a positive effect on whether older adults participate in PA. In this study, the mean age of the group observed was 68.64 ± 7.43 years, and higher education was related to more time spent in PA. Earlier studies showed that high physical activity is related to a higher educational level (45, 46). Individuals with high educational achievement are more likely to tolerate and be open to diverse perspectives and to assimilate external information constructively (47). People with higher education recognize to a greater extent the importance of nutrition, diet, and the role of physical activity in treating their disease (48). Research has shown that education affects personal self-control, which affects PA and even health. There is a positive mediating effect of self-reported PA on the relationship between personal control and health when an individual's educational level is high, and there is a negative mediating effect of self-reported PA when an individual's educational level is low (49). Therefore, educational differences may have an effect on the results of PA in elderly populations.

### Limitations

First, given the self-reported nature of the questionnaire, it is possible that reporting bias led to some data distortion. To better meet social expectations, patients with chronic diseases may report values that are longer than the actual PA time. Second, because this study was cross-sectional, the results of this study only showed that at the time point considered, the diagnosis group spent more time on PA than the other two groups. The correlation between diagnosis and PA needs to be further explored. Finally, the proportion and time of older adults participating in recreational activities were the lowest. Encouraging the participation of older adults with chronic diseases is worth further exploration. We will continue to explore this direction in future research.

## Conclusions

In conclusion, our results of representative data from Shanghai showed that elderly people generally participated more in transportation and activities at housework. A diagnosis of chronic disease was associated with time spent on PA and PA MET-minutes. This finding suggests that the possibility for older adults, especially those with abnormal health indicators, to participate in PA should be considered in the prevention and control of chronic diseases. When carrying out community-based chronic disease prevention in Shanghai that promotes PA for elderly people, priority can be given to recommending moderate-intensity housework or transportation (such as walking or cycling), which are preferred by elderly people. More research is needed to understand the mechanisms of diagnostic information on the participation in PA among residents with chronic diseases.

## Data Availability Statement

The original contributions presented in the study are included in the article/supplementary materials, further inquiries can be directed to the corresponding authors.

## Ethics Statement

The studies involving human participants were reviewed and approved by the Research Ethics Committee of the Tongji University. The patients/participants provided their written informed consent to participate in this study.

## Author Contributions

XH, WZ, JS, and ZW designed the study. XH, DY, HJ, and WY conduct literature analysis. JH, XG, XH, and JS conducted the data analysis, YL, LZ, JH, and JS made all tables. YY, NC, and XH produced all figures. DY, JS, and ZW guarantee access to research resources. All authors participated in the writing, revision, final review of the manuscript, read, and approved the final manuscript.

## Funding

This study was supported by the Shanghai Education Science Research Project (Grant Number: C2021039). Data extraction was financially funded by the Natural Science Foundation of China (Grant Numbers: 71774116 and 71603182). The analysis and interpretation of the data guided by the statisticians were funded by the grants from the Shanghai Public Health Outstanding Young Personnel Training Program (Grant Number: GWV-10.2-XD07). The writing and revision, including the language improvement, were sponsored by Shanghai Pujiang Program (Grant Number: 2019PJC072), and Shanghai Leading Talents (Grant Number: YDH-20170627). The research presented in this article is that of the authors and does not reflect the official policy of the NHC.

## Conflict of Interest

The authors declare that the research was conducted in the absence of any commercial or financial relationships that could be construed as a potential conflict of interest.

## Publisher's Note

All claims expressed in this article are solely those of the authors and do not necessarily represent those of their affiliated organizations, or those of the publisher, the editors and the reviewers. Any product that may be evaluated in this article, or claim that may be made by its manufacturer, is not guaranteed or endorsed by the publisher.

## Abbreviations

PA: Physical activity
NCDs: Non-communicable diseases
WHO: World Health Organization
BMI: Body mass index
TC: Total cholesterol
HDL: High-density lipoprotein
LDL: Low-density lipoprotein
TG: Triglycerides
OR: Odds Ratios.
